# Transvaginal single-port versus multi-port laparoscopic sacrocolpopexy: a retrospective cohort study

**DOI:** 10.1186/s12893-022-01535-w

**Published:** 2022-03-04

**Authors:** Junwei Li, Yizhen Sima, Changdong Hu, Xiaojuan Wang, Zhiying Lu, Keqin Hua, Yisong Chen

**Affiliations:** Department of Gynecology, Obstetrics and Gynecology Hospital of Fudan University, 128 Shenyang Road, Shanghai, 200090 China

**Keywords:** Transvaginal single-port laparoscopy, Sacrocolpopexy, Pelvic organ prolapse, Mesh

## Abstract

**Background:**

Sacrocolpopexy is the gold standard treatment for apical prolapse. With the development of minimally invasive surgical techniques, the new approach of transvaginal single-port laparoscopic sacrocolpopexy (TS-LSC) has become available. However, its therapeutic effects remain unclear. The aim of this study is to compare the middle-term clinical outcomes of transvaginal single-port laparoscopic sacrocolpopexy with multi-port laparoscopic sacrocolpopexy (LSC) for apical prolapse.

**Methods:**

We conducted a retrospective cohort study. Patients with advanced apical prolapse who underwent either TS-LSC or LSC between May 2017 to June 2019 were enrolled. Baseline demographics, perioperative results, perioperative and postoperative complications, pelvic organ prolapse quantification (POPQ) scores, pelvic floor distress inventory (PFDI-20) score and pelvic organ prolapse/urinary incontinence sexual function questionnaire (PISQ-12) score were collected at 2 years.

**Results:**

89 subjects were analyzed: 46 in TS-LSC and 43 in LSC group. Follow-up time was 38.67 ± 7.46 vs 41.81 ± 7.13 months, respectively. Baseline characteristics and perioperative outcomes were similar except that pain score was lower (2.37 ± 0.90 vs 3.74 ± 1.05) and cosmetic score was higher (9.02 ± 0.75 vs 7.21 ± 0.89) in TS-LSC group (P < 0.05). Complication rates did not differ between groups. 3 mesh exposure in each group were noted. Recurrence rate was 2.17% in TS-LSC and 6.98% in LSC, no apical recurrence occurred. Constipation was the most common postoperative symptom. Besides, patients in TS-LSC group had better POP-Q C point (− 6.83 ± 0.54 vs − 6.39 ± 0.62, P < 0.05), and similar Aa, Ap and TVL values. Bladder and pelvic symptoms were improved in both groups, but colorectal symptoms were not relieved. There were no differences of PISQ-12 scores between groups.

**Conclusion:**

TS-LSC was not inferior to LSC at 2 years. Patients may benefit from its mild pain, better cosmetic effect and better apical support as well as good safety and efficacy. TS-LSC is a promising considerable choice for advanced vaginal apical prolapse.

*Trial registration* ChiCTR2000032334, 2020-4-26 (retrospectively registered)

## Background

Pelvic organ prolapse (POP) occurs when the pelvic floor is partially or wholly descended due to the defect of pelvic supportive tissues. Women have a 20% lifetime risk of undergoing POP surgeries [1], the focus of which is to restore normal anatomy and preserve function [2]. A pivotal role of Level I (apical) support has been demonstrated [3], and many procedures have been described, by vaginal, abdominal or laparoscopic approach. Sacrocolpopexy is a procedure designed to treat uterine or vault prolapse, considered to be the preferred and gold standard surgery for the repair of vaginal apical prolapse [4]. Laparoscopic sacrocolpopexy (LSC) is recognized as being equivalent to abdominal sacrocolpopexy (ASC) and equivalent or superior to transvaginal vaginal mesh (TVM) and other surgical procedures [5, 6]. However, there are inevitably 4–5 wounds in the abdominal wall, and the estimated rate of trocar site hernia is 0.8–2.9%, even up to 27.6% [7].

In recent years, minimally invasive surgeries have been dramatically developed, transvaginal single-port laparoscopy can provide better visibility and operative precision than vaginal approach, less wound-related complications and better cosmetic results than conventional laparoscopic approach, perfectly meets the criterion of minimally invasive surgery and can be a helpful tool for urogynecologic surgeons [8]. Transvaginal single-port laparoscopic sacrocolpopexy was first reported by our team [9], and had been carried out as a routine procedure in our hospital. As a novel procedure, there is limited data to describe the outcomes. Liu [10] reported short-term outcomes of 26 cases, showing significant improvements in both physical prolapse and quality of life, with no complications of mesh exposure, pain, hematoma, infection, or de novo urinary incontinence. There was a paucity of data of longer-term clinical outcomes or comparison with other procedures. The aim of this study is to assess the middle term clinical outcomes of transvaginal single-port laparoscopic sacrocolpopexy (TS-LSC) compared with conventional laparoscopic sacrocolpopexy (LSC).

## Methods

### Study design and participants

This study was conducted at the Obstetrics and Gynecology Hospital of Fudan University. This study was approved by the Research Ethics Committee of the hospital (No 2017-90).

A retrospective cohort study was conducted for patients who underwent either TS-LSC or LSC from May 2017 to June 2019. Laparoscopic or transvaginal single-port laparoscopic sacrocolpopexy was indicated for apical prolapse in patients of 45–75 years old who were POPQ ≥ stage III, or symptomatically ≥ stage II, or recurrent POP ≥ stage II. Apical prolapse might accompany with mild to moderate anterior or posterior prolapse. Uterine size or concurrent procedures such as hysterectomy, colporrhaphy, adnexal surgery, or incontinence surgery did not preclude enrollment. When adnexal mass was found either in ultrasound or intraoperative exploration, adnexal surgeries were performed. Preventive salpingo-oophorectomy might be performed after informed consent for post-menopause patients. Exclusion criteria included malignant tumor, dementia, and inflammatory bowel disease.

The electronic medical records were reviewed to collect data on demographics, physical examination, medical and surgical histories, concomitant procedures, mesh type, perioperative complications, and follow-up data. All patients were clinical followed-up at 3 months, 1 year, 2 years. Physical examinations included a pelvic organ prolapse quantification (POP-Q) examination were performed. Symptoms (such as constipation, urinary incontinence) and complications (such as mesh erosion, frequent urinary tract infection) were also recorded. Pelvic floor distress inventory-short form 20 (PFDI-20) and pelvic organ prolapse/urinary incontinence sexual function questionnaire (PISQ-12) were assessed for quality of life before surgery and at follow-up time, by mail, or phone or print-out copy. Visual analogue scale (VAS) pain score was recorded 24 h after surgery. Cosmetic score was recorded before discharge [11]. The cosmetic evaluation was conducted by the patients using the VAS, from 0 points = very unsatisfied to 10 points = very satisfied. POP-Q ≥ stage II was defined as recurrence [12] and Stage 0 or Stage 1 of POPQ was defined as objective success. Subjective success was defined as 0 point based on the PFDI-20 question 3. Constipation was defined as ≥ 3 point based on PFDI-20 question 7. Morbidity was defined as temperature ≥ 38 °C for more than 2 times in an interval of at least 4 h.

### Surgical technique

Patients were administered general anesthesia and placed in the lithotomy, received a Foley urinary catheter, and the perineum and vagina were sterilized. No bowel preparation was needed before the surgery. A 30° laparoscope was used during surgery. The energy source was an ultrasound knife (Harmonic) and a 10 mm LigaSure vessel sealing system (Covidien, Valleylab). LigaSure was used to cut off the uterine vessels and ovary vessels. The ultrasound knife was used for precise manipulations such as separation of vesicovaginal/rectovaginal space, incision of pelvic peritoneum and exposure of longitudinal ligament. A type I microporous polypropylene mesh was used according to the preference of the surgeon. Prophylactic antibiotics of cefuroxime and metronidazole were given for 48 h.

### Transvaginal single-port laparoscopic sacrocolpopexy (TS-LSC)

**Fig. 1:**
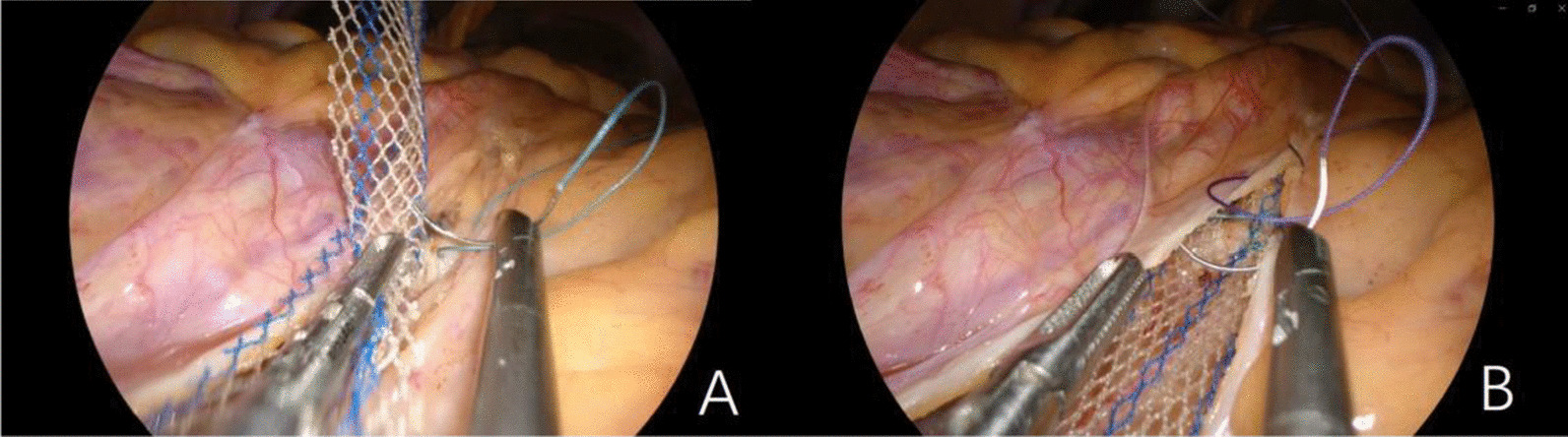
Critical steps of transvaginal single-port laparoscopic sacrocolpopexy. **A** The long arm of Y-shaped mesh was anchored to the longitudinal ligament lying on S1 vertebrae. **B** The peritoneum was closed

The procedures were described in the previous video article [9]. Lengthened instruments and laparoscope were used. After the hysterectomy was done,  the single-port device was established. The right side of the pelvic peritoneum was opened, from sacral promontory to the vaginal cuff. Then the Y-shaped mesh was clipped according to the length of vaginal wall. The posterior vaginal wall was separated with the help of phenylephrine hydrochloride-methylene blue water cushion. The posterior arm of the mesh was sutured with the vaginal wall, then the mesh was flatted by laparoscopy with the junction of the mesh located at the vaginal cuff. The long arm of mesh was anchored to the longitudinal ligament lying on S1 vertebrae with tension-free, using Ethibond, non-absorbable, synthetic and multifilament sutures from Ethicon (seeing in Fig. 1A). Cut off the redundant part of the mesh, and close the peritoneum (seeing in Fig. 1B). The separated anterior vaginal wall was sutured to the anterior arm of the mesh. The vaginal cuff was closed. Perineal body repair was performed at the discretion of the surgeon when the width of the vaginal orifice was more than 3 fingers at the end of the procedure. Lodophor gauze was inserted into the vagina for 24 h.

### Laparoscopic sacrocolpopexy (LSC)

Laparoscopic sacrocolpopexy was performed as Coolen reported [13], with four trocars, one for the laparoscope and three side trocars. The peritoneum from the promontory to the vault was incised to expose the rectovaginal and vesicovaginal fascia, extending to the sacral promontory. The anterior arm of the Y-shaped mesh was attached between the vagina and the bladder anteriorly, and the posterior arm was fixed with the posterior vaginal wall. The long arm of mesh was anchored to the same position as TS-LSC, at the longitudinal ligament lying on S1 vertebrae. The mesh was reperitonealised. Anterior/posterior midline colporrhaphy was performed when Ba/Bp > 0 cm.

### Statistical analysis

Continuous variables were presented as Mean ± SD, and discrete variables, were presented as case number and percentage. Chi-square test and t test were calculated using SPSS 19.0. A 2-sided P value < 0.05 was considered statistically significant.

## Results

50 patients underwent TS-LSC and another 48 patients underwent LSC from May 2017 to June 2019. One died, one got dementia after surgery and two failed to follow up in TS-LSC group, while five failed to follow up in LSC group. There were 46 subjects in TS-LSC group (92.0%) and 43 subjects in LSC group (89.6%) in the analysis. As shown in Table 1, baseline characteristics were similar between the two groups. Follow-up time was 38.67 ± 7.46 months in TS-LSC group vs 41.81 ± 7.13 months in LSC group. All subjects had stage II or greater apical prolapse, accompanied by mild-to-moderate anterior or posterior compartment prolapse.

**Table 1:** Baseline characteristics

Variables	TS-LSC (n = 46)	LSC (n = 43)	P
Age (year)	54.98 ± 6.55	56.70 ± 7.37	0.25
BMI (kg/m^2^)	23.65 ± 2.39	24.17 ± 2.09	0.27
Parity	1.35 ± 0.53	1.37 ± 0.82	0.87
Menopause	33 (71.74)	27 (62.79)	0.37
Diabetes	1 (2.17)	2 (4.65)	0.51
Previous abdominal surgeries	15 (32.61)	9 (20.93)	0.22
Previous POP surgeries	2 (4.35)	0 (0.00)	0.18
Follow up time (months)	38.67 ± 7.46	41.81 ± 7.13	0.05
Prolapse stage			
Anterior	2.17 ± 0.71	2.40 ± 0.73	0.15
Apical	2.80 ± 0.58	2.70 ± 0.67	0.43
Posterior	1.20 ± 1.00	1.37 ± 0.95	0.40
POP score			
Aa	0.00 ± 1.38	0.63 ± 1.73	0.06
C	2.20 ± 2.00	1.97 ± 2.27	0.61
Ap	− 1.82 ± 1.46	− 1.29 ± 1.77	0.13
TVL	7.33 ± 0.56	7.26 ± 0.53	0.54

Table 2 shows the perioperative results. Colporrhaphy was more commonly performed in the TS-LSC group, and adnexal surgeries were more performed in LSC group, while the other concomitant procedures were similar. There were 19 adnexal surgeries in TS-LSC group, 9 therapeutically and 10 preventively, while 31 adnexal surgeries were performed in TS-LSC group, 11 therapeutically and 20 preventively. The type of mesh in the two groups was different. TiLOOP Mesh was the most common in TS-LSC, but Dyna mesh was significantly more than the other two types of mesh in LSC group. All the surgeries are eventful, and there was no conversion to laparotomy or conventional multiple port laparoscopy. There were no differences of estimated blood loss, operative time, hospital costs, postoperative hospital stay, or morbidity between two groups. However, the pain score was lower (2.37 ± 0.90 vs 3.74 ± 1.05) and cosmetic score was higher (9.02 ± 0.75 vs 7.21 ± 0.89) in TS-LSC group (P = 0.00).

**Table 2:** Perioperative results

Variables	TS-LSC (n = 46)	LSC (n = 43)	P
Concomitant procedures			
Hysterectomy	43 (93.48)	43 (100.00)	0.09
Colporrhaphy	27 (58.70)	9 (20.93)	0.00*
Adnexal surgery	19 (41.30)	31 (72.09)	0.00*
Incontinence surgery	5 (10.87)	2 (4.65)	0.28
Conversion to open or multiport laparoscopy	0 (0)	0 (0)	1.00
Mesh type			0.00
Artisyn Y	21	13	
Dyna mesh PR	0	28	
TiLOOP Mesh	25	2	
Estimated blood loss (ml)	95.43 ± 54.60	96.98 ± 50.64	0.89
Operative time (ml)	134.50 ± 32.21	132.67 ± 40.41	0.81
Postoperative stay (day)	6.00 ± 2.10	5.42 ± 1.40	0.13
Hospital costs (USD)	4427.11 ± 1284.57	4280.29 ± 984.84	0.55
Morbidity	16 (34.78)	15 (34.88)	0.99
VAS pain score	2.37 ± 0.90	3.74 ± 1.05	0.00*
VAS cosmetic score	9.02 ± 0.75	7.21 ± 0.89	0.00*

^*^P < 0.05

Regarding perioperative complications (Table 3), no injury, blood transfusion, hematoma or bowel obstruction occurred. One patient (2.17%) in TS-LSC group required re-admission within 7 days due to postoperative fever. Antibiotics were administrated because of detection of E coli in vaginal culture, and the patient was discharged after 6 days. One patient (2.33%) in LSC group presented with shortness of breath and was found pulmonary artery embolism 3 days after surgery, who were treated with thrombolytic therapy. Regarding postoperative complications, 3 mesh exposure in each group were noted. In TS-LSC group, 2 exposed meshes were treated conservatively, and 1 was reoperated to remove the exposure part. In LSC group, one was treated conservatively with estrogen ointment, one was reoperated to partly remove the mesh, and the other was reoperated to treat vesico-vaginal fistula. No patient experienced apical recurrence. There were one recurrent stage II posterior prolapse in TS-LSC group and three recurrent stage II anterior prolapse in LSC group, Objective success rate was 97.83% in TS-LSC group vs 93.02% in LSC group and subjective success rate was 100% vs 90.70%, respectively. No recurrent patients received a second pop operation. One patient in TS-LSC group required a TVT-A due to de novo SUI. Constipation was the most common postoperative symptom, affecting 4.35% of TS-LSC and 6.98% of LSC patients.

**Table 3:** Complications

N (%)	TS-LSC	LSC	P
Perioperative complications			
Intraoperative injury	0 (0.00)	0 (0.00)	1.00
Hematoma	0 (0.00)	0 (0.00)	1.00
Blood transfusion	0 (0.00)	0 (0.00)	1.00
Bowel obstruction	0 (0.00)	0 (0.00)	1.00
Pulmonary artery embolism	0 (0.00)	1 (2.33)	0.30
Re-admission within 7 days	1 (2.17)	0 (0.00)	0.33
Postoperative complications			
Mesh exposure	3 (6.52)	3 (6.98)	0.93
De novo SUI	1 (2.17)	0 (0.00)	0.33
Constipation	2 (4.35)	3 (6.98)	0.59
Urinary tract infection	2 (4.35)	0 (0.00)	0.17
Dyspareunia	2 (4.35)	1 (2.33)	0.60
Reoperation	2 (4.35)	2 (4.65)	0.95
Recurrence	1 (2.17)	3 (6.98)	0.27

We compared POP-Q between two groups at 2 years after surgery (Table 4). There was significant improvement in all POP-Q scores from baseline to 2 years for both groups. TVL was not shortened after surgery. Compared with LSC group, patients in TS-LSC group had similar Aa, Ap and TVL values, but better C point (− 6.83 ± 0.54 vs − 6.39 ± 0.62, P < 0.05).

**Table 4:** Anatomic changes

POPQ	TS-LSC	LSC	P
Aa			
Pre-op	0.00 ± 1.38	0.63 ± 1.73	0.06
2 years post-op	− 2.86 ± 0.35	− 2.58 ± 0.76	0.08
P	0.00*	0.00*	
C			
Pre-op	2.20 ± 2.00	1.97 ± 2.27	0.61
2 years post-op	− 6.83 ± 0.54	− 6.39 ± 0.62	0.01*
P	0.00*	0.00*	
Ap			
Pre-op	− 1.82 ± 1.46	− 1.29 ± 1.77	0.13
2 years post-op	− 2.86 ± 0.44	− 2.74 ± 0.43	0.30
P	0.00*	0.00*	
TVL			
Pre-op	7.33 ± 0.56	7.26 ± 0.53	0.54
2 years post-op	7.41 ± 0.57	7.29 ± 0.46	0.36
P	0.51	0.77	

^*^P < 0.05

PFDI-20 scores, POPDI-6 scores and UDI-6 scores were significantly improved 2 years after surgery in both groups (P = 0.00) (Table 5), which were similar between groups. However, there were no differences of CRADI scores at 2 years follow-up when compared with pre-operation, no matter TS-LSC group or LSC group. Colorectal symptoms were not relieved, despite the significant improvement of bladder and pelvic symptoms. Sexual function was preserved in both groups with the equivalent PSIQ-12 scores after surgery.

**Table 5:** Quality of life

Questionnaires	TS-LSC	LSC	P
PFDI-20			
Pre-op	57.77 ± 23.32	58.48 ± 28.80	0.90
2 years post-op	22.42 ± 24.70	23.69 ± 27.68	0.83
P	0.00*	0.00*	
POPDI-6			
Pre-op	30.07 ± 14.77	24.52 ± 13.59	0.07
2 years post-op	8.73 ± 10.53	8.93 ± 13.19	0.94
P	0.00*	0.00*	
CRADI-8			
Pre-op	4.42 ± 5.00	7.12 ± 11.36	0.15
2 years post-op	3.87 ± 6.72	4.09 ± 7.70	0.89
P	0.66	0.18	
UDI-6			
Pre-op	23.28 ± 13.62	26.40 ± 15.24	0.31
2 years post-op	9.82 ± 13.86	10.91 ± 13.27	0.71
P	0.00*	0.00*	
PISQ-12			
Pre-op	34.14 ± 6.59	36.57 ± 4.99	0.06
2 years post-op	35.74 ± 4.38	35.47 ± 6.01	0.82
P	0.20	0.36	

*PFDI-20* pelvic floor distress inventory-short form 20, *POPDI-6* pelvic organ prolapse distress inventory 6, *CRADI-8* colorectal–anal distress inventory 8, *UDI-6* urinary distress inventory 6, *PISQ-12* pelvic organ prolapse/urinary incontinence sexual function questionnaire

^*^P < 0.05

## Discussion

Sacrocolpopexy is the preferred procedure for apical vaginal prolapse, and laparoscopic seems to be the preferred approach to sacrocolpopexy [14]. Laparoscopic sacrocolpopexy (LSC) has been widely carried out. Thanks to the rapid development of minimally invasive techniques, laparoscopic single-site surgery (LESS), natural orifice transvaginal endoscopy (vNOTES), even robotic assisted single-site surgeries have been performed. To our knowledge, this is the first study to compare transvaginal single-port laparoscopic sacrocolpopexy (TS-LSC) with other procedures, and being followed up for more than 2 years.

In this present study, TS-LSC was not inferior to LSC at 2 years follow-up. Both procedures showed good subject and objective outcomes, significantly corrected the apical prolapse and improved quality of life, accompanied by low rates of mesh-related complications and recurrence. In addition, TS-LSC seemed to be superior in good cosmetics, mild pain and ideal apical anatomic correction. No apical recurrence occurred, and C point in TS-LSC was − 6.81 ± 0.56, significant better than that of LSC group (− 6.48 ± 0.60), which was the evidence of its adequate Level I support. The direction of mesh implantation in TS-LSC was from vagina to pelvic cavity, with posterior arm first, long arm next, and the anterior arm at last, which was the opposite with LSC. It was easier to adjust the length and tightness of mesh. In order to reduce the mesh volume at vaginal cuff, the junction of the mesh was usually a little higher than the cuff. When the vaginal cuff was closed transvaginally, it was fixed with posterior arm of mesh with one absorbable suture. As a result, the C point (vaginal cuff) was not totally mobilizable, not prone to descend again. This might explain the 0.5 cm difference of C point between TS-LSC and LSC.

There was no anterior compartment recurrence in TS-LSC group, but there were 3 in LSC. Prolapse recurrence in the anterior and posterior compartments may be due to a more challenging caudad dissection during laparoscopic sacrocolpopexy, which is often limited by poor tissue-plane separation and bleeding [14]. Wong’s [15] study demonstrated that prolapse recurrence seemed to be related to mesh position and mobility. The more distal the mesh was placed in the anterior compartment, the less likely it was for prolapse to recur in the anterior compartment. For every mm that the mesh is placed closer to the bladder neck, the risk of prolapse recurrence in the anterior compartment on clinical examination was reduced by 6% and on ultrasound by 7%. The separation of vaginal wall and fixation of anterior/posterior arm of the mesh through laparoscopic approach were absolutely the technical difficulty of LSC. But in TS-LSC procedures, the rectovaginal and vesicovaginal spaces was exposed with the help of phenylephrine hydrochloride-methylene blue water cushion, which made this difficulty much easier, the tissue-plane separation was more precise with the guide of blue color, and bleeding was relatively less. The anterior and posterior part of the Y shaped mesh was placed in direct vision, the mesh could be placed much lower, even to the hymen. Invisible abdominal wound in transvaginal single-port laparoscopy leads to better cosmetics results and less postoperative pain in TS-LSC, which were in accord with other studies [16, 17].

Transvaginal single-port laparoscopy could provide better visibility and operative precision, also a less degree of triangulation loss and instrument crowding than LESS due to vaginal elasticity [18]. Despite all these advantages, surgeons need systematic skill practice of both vaginal and laparoscopic surgeries, readjusting the opposite surgical field and direction, and paying more attention to teamwork. Permanent surgery staff and quick switching of positions and devices are quite helpful [16]. Operative time of transvaginal single-port laparoscopic pelvic reconstruction with Y mesh, which was derived from TS-LSC, significantly declined after 45 cases [17]. Learning curve of TS-LSC seems not to be longer than LSC. There is no doubt that quality of LSC/TS-LSC improves with experience and structured learning. The two surgeons performing TS-LSC both have an experience of POP repair and laparoscopic surgeries for more than 10 years. These 46 patients in the study were the first early cases of TS-LSC in our hospital, and the number of cases has been reached to nearly 250 in our 4 year experience. Along with the maturation of the surgical skills, operative time and postoperative hospital stay are getting shorter. It is reasonable to infer that TS-LSC may have even better clinical outcomes. It is noteworthy that mesh-augmentation and relatively longer time of transvaginal operation may lead to potential risk of infection and mesh-related complications. In this study, there was no difference of postoperative morbidity between two groups. We used povidone iodine washing before vaginal wound closing in TS-LSC group. We also suggest that antibiogram of prophylactic antibiotics should cover the common vaginal bacteria of *Escherichia* *coli*, *Enterococcus faecalis*, and adjust the medication plan according to vaginal cultures in time.

Quality of life was significantly improved after surgery, with the PFDI-20 scores, POPDI-6 scores and UDI-6 scores decreased markedly 2 years after surgery in both groups (P = 0.00). However, there were no differences of CRADI scores after surgery, which suggested that the bowel function was not improved. Constipation was the most common postoperative symptoms. Forsgren [19] and Crane [20] also came to the same conclusion. POP patients usually have common risk factors such as neuropathic and muscular injury to the pelvic floor after vaginal delivery and the effects of aging. Obstructive bowel symptoms are significantly associated with the presence of prolapse [21], and sacrocolpopexy is also associated with obstructed defecation. Sacrocolpopexy may involve an overcorrection of the distal anterior rectal wall, the operation could also interfere with the complex dynamic pressure mechanisms involved in regulating anal closure. Interruption of distal nerve branches to the anal sphincter complex at vaginal dissection may also provide the grounds for rectal emptying difficulties. So the colorectal symptoms could not be significantly improved. Ramanah [22] recommended that vaginal perineorrhaphy instead of posterior repair with mesh could be advocated and the patient should be informed of the potential risk of de novo anorectal symptoms.

The postoperative hospital stay was 5–6 days in our study, which was longer than western countries. This could be complained by the differences of medical insurance systems. Most of the hospital cost of our patients could be covered by medical insurance even the mesh. The patients were willing to stay in the hospital for a relative longer period until they were better recovered both physically and psychologically. We also had more sufficient time to observe the postoperative recovery process and any abnormal conditions could be dealt with timely. In recent cases, the postoperative hospital stay has been shortened for 1–2 days because of the ERAS development and technical maturity.

Since the US Food and Drug Administration (FDA) stopped the distribution of transvaginal mesh, native tissue repair surgeries have been reevaluated. Shull’s technique of uterosacral ligament suspension (USLS) provides a safe and effective technique for apical prolapse without prosthetic materials. However, USLS resulted in a higher prolapse recurrence rate than sacrocolpopexy for Stage III prolapse [23] and the risk of ureteral obstruction could not be totally avoided even if intraoperative cystoscopy was performed [24]. Sacrocolpopexy seems to be superior in advanced apical prolapse. In order to reduce recurrence and complications, our surgical team are trying to combine SC and USLS together, performing a new native tissue repair procedure, randomized clinical trial is ongoing.

The main limitation of our study is that it is a retrospective cohort study and there is a possibility of selection bias. The sample size of the study is relatively small. As a novel procedure, this study was designed to assess the middle-term outcomes of at least 2 years, clinical data available for analysis was limited. In addition, surgeons, the types of mesh and concomitant procedures in the two groups were different which might lead to different outcomes. There were only two surgeons performing TS-LSC, while the other surgeons in our hospital performed LSC. The discretion and operation habits were different. Surgeons in LSC group tended to choose Dyna mesh and perform anterior/posterior midline colporrhaphy when Ba/Bp > 0 cm. But surgeons in TS-LSC preferred to choose the longer Artisyn Y mesh instead of the shorter TiLoop mesh, putting the mesh lower to deal with the anterior/posterior prolase. Besides, perineal body repair was performed when the width of the vaginal orifice was more than 3 fingers. Future researches involving prospective randomized control trials would provide strong evidence.

## Conclusion

Transvaginal single-port laparoscopic sacrocolpopexy (TS-LSC) was not inferior to laparoscopic sacrocolpopexy at 2 years follow-up. Patients may benefit from its mild pain, better cosmetic effect and better apical support as well as good safety and efficacy. TS-LSC is a promising considerable choice for apical prolapse. However, the functional and anatomical results must be further determined over a longer term before definitive conclusions can be drawn. Studies of long follow-up time and large scale are still needed.

## Data Availability

The datasets used and analyzed during the current study available from the corresponding author on reasonable request.

## Abbreviations

TS-LSC: Transvaginal single-port laparoscopic sacrocolpopexy
LSC: Laparoscopic sacrocolpopexy
SC: Sacrocolpopexy
PFDI-20: Pelvic floor distress inventory-20
PISQ-12: Pelvic organ prolapse/urinary incontinence sexual function questionnaire-12
POP-Q: Pelvic organ prolapse quantification
VAS: Visual analogue scale
POP: Pelvic organ prolapse
POPDI-6: Pelvic organ prolapse distress inventory-6
CRADI-8: Colorectal-anal distress inventory-8
UDI-6: Urinary distress inventory-6
BMI: Body mass index
FDA: Food and Drug Administration
USLS: Uterosacral ligament suspension
